# HMGA2-Rearranged Lipoblastoma in an Adult Patient With Novel GRIP1 Gene Fusion Partner

**DOI:** 10.7759/cureus.115291

**Published:** 2026-08-27

**Authors:** Rana Naous, Aaron Victor

**Affiliations:** 1 Pathology, The Ohio State University Wexner Medical Center, Columbus, USA

**Keywords:** adult, gene rearrangement, grip1, hmga2, lipoblastoma

## Abstract

Lipoblastoma is a benign tumor of adipocytic differentiation originating from embryonal white fat that is commonly described in young children, with rare cases arising in adults.* PLAG1* gene (8q11-13) rearrangement is a characteristic feature of lipoblastoma. However, *HMGA2 *gene rearrangement has also been reported in a few cases of lipoblastoma, with the gene fusion partners reported thus far being *EP400*, *FGD6, *and* GSN *genes. Herein, we report an *HMGA2*-rearranged lipoblastoma arising in the chin of a 51-year-old female with a novel gene fusion partner, the* GRIP1* gene, that has not been previously described in lipoblastomas. Our case adds to the growing pool of gene fusion partners of *HMGA2*-rearranged lipoblastomas and expands the molecular spectrum of lipoblastomas in general beyond the previously described *PLAG1 *gene rearrangements, thereby suggesting that lipoblastomas may be more molecularly heterogeneous than previously appreciated.

## Introduction

Lipoblastoma is a benign lipogenic neoplasm of embryonal white fat origin with a common incidence in young children, usually younger than five years old, slight male predominance, and predilection for the trunk and extremities, though other sites have been involved, including the mediastinum, retroperitoneum, or viscera [1]. Lipoblastoma cases have been reported in adults [2]. *PLAG1* gene (8q11-13) rearrangement is a characteristic feature of lipoblastoma [3]. However, *HMGA2* gene rearrangement has also been reported in a few cases of lipoblastoma, with the gene fusion partners reported thus far being *EP400*, *FGD6*, and *GSN* genes [4-6].

We present a rare case of *HMGA2*-rearranged lipoblastoma in the chin of a 51-year-old female, characterized by a novel gene fusion partner, the *GRIP1* gene, not previously documented in lipoblastomas.

## Case presentation

**Figure 1:**
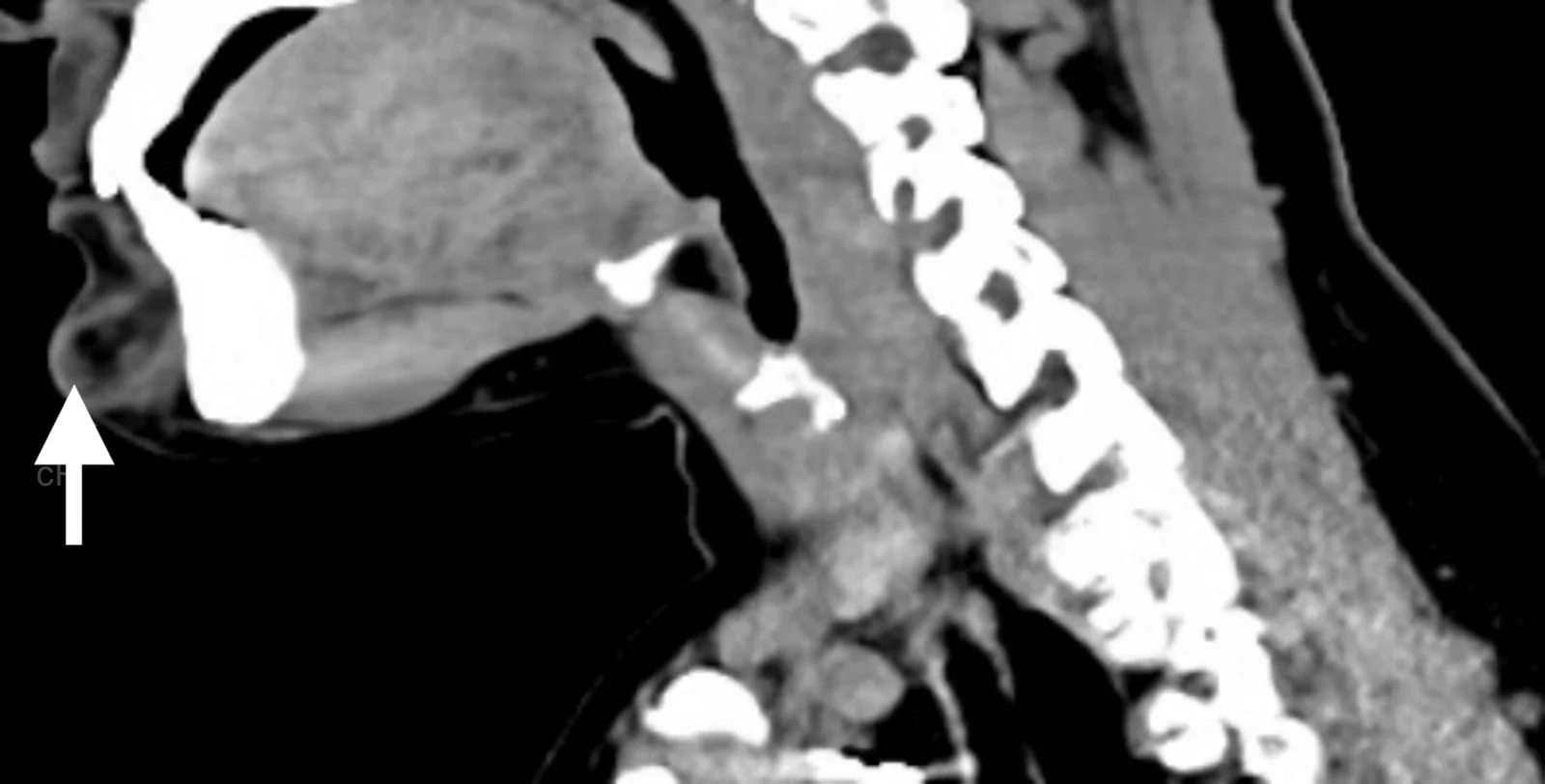
CT of the neck CT neck illustrating a small nodule within the subcutaneous tissue of the right chin (arrow).

The patient is a 51-year-old African American female with a history of heart transplant on chronic immunosuppression (tacrolimus, mycophenolate, and sirolimus), deep vein thrombosis (DVT), type 2 diabetes mellitus, and iron deficiency anemia who presented to her primary care physician for a chief complaint of a nodule on her chin that has been present since 2023 and has recently grown in size in the last year. A CT of the neck (Figure 1) was performed and showed an indeterminate small (<4 cm) subcutaneous mass in the right chin.

**Figure 2:**
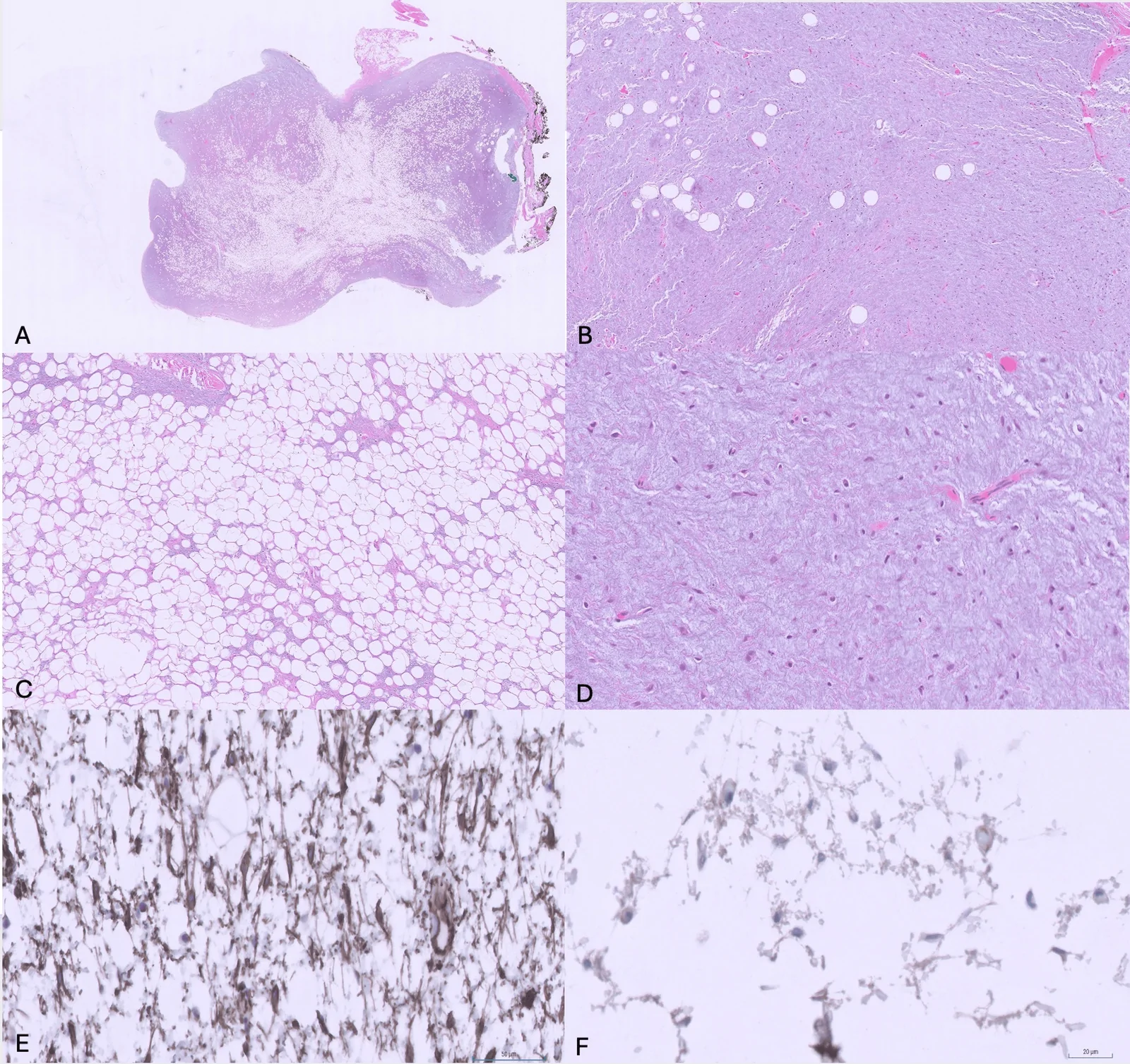
HMGA2::GRIP1 fusion-positive lipoblastoma (A) Low-power examination demonstrating a well-circumscribed lipogenic neoplasm with zonation, whereby the mature adipocytes are in the center and the spindle cells are more towards the periphery (H&E, 0.25×). (B-C) Scattered spindle cells and mature adipocytes are seen within a myxoid stroma with increased vascularity (H&E, 5×). Higher-power examination showing the bland nature of the spindle cell component (H&E, 20×).

The clinical decision was to entirely excise the nodule in a marginal fashion. Microscopic examination revealed a relatively well-circumscribed lipogenic neoplasm (Figure 2A) comprised of scattered minimally atypical spindle cells and mature adipocytes set in a myxoid stroma with an increase in vascularity (Figures 2B-D). The neoplasm showed a zonation pattern whereby the mature adipocytes were noted more towards the center of the lesion and the spindle cells more towards the periphery. There was no evidence of frank atypia or necrosis, and mitotic figures were difficult to find. Immunohistochemical stains were positive for CD34 (Figure 2E) and variably positive for desmin (Figure 2F), while *STAT6* and SMA were all essentially negative. FISH for *MDM2* gene amplification was negative. Although the tumor focally abutted the inked margin of resection, it was completely excised.

**Figure 3:**
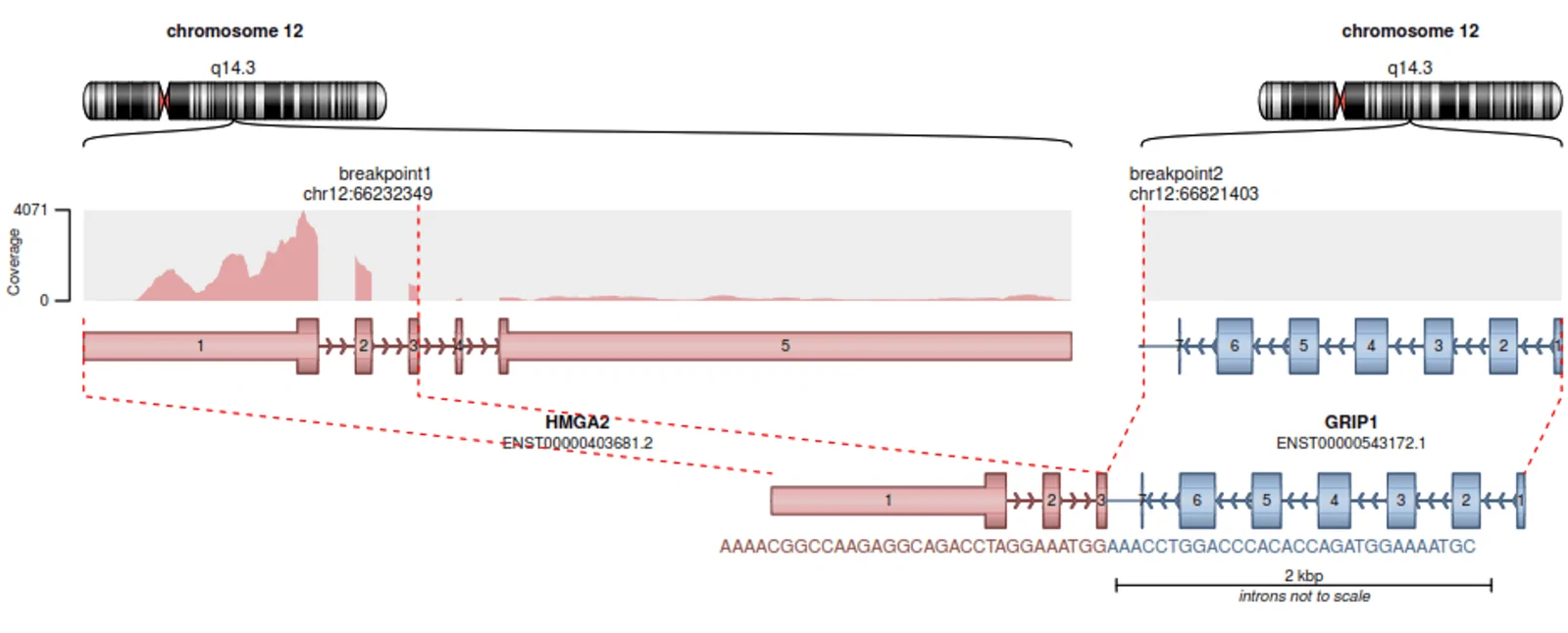
Targeted RNA sequencing The HMGA2::GRIP1 fusion detected by Targeted RNA Sequencing was annotated using transcripts ENST00000403681.2 (*HMGA2*) and ENST00000543172.1 (*GRIP1*). This gene fusion entails loss of *HMGA2* C-terminal regulatory domains, leading to a gain of function. In contrast, the translocation partner gene *GRIP1* is fused in the reverse orientation, resulting in an untranslated out-of-frame product rather than a functional chimeric protein. Image credit: Dr. Aaron Victor using the software draw_fusions.R from Arriba 2.5.1 (German Cancer Research Center (DKFZ), Heidelberg, Germany).

RNA sequencing for fusion detection was performed on tumor-derived RNA using a hybrid-capture-based assay targeting the full exon regions of 230 genes recurrently involved in solid tumor fusions. Library preparation included ribosomal RNA depletion, cDNA synthesis, adapter ligation, and PCR amplification, followed by hybrid capture and sequencing on an Illumina platform. Sequence alignment and quality metrics were generated using the Illumina BaseSpace pipeline and SAMtools, and fusion detection was performed with the splice-aware algorithm Arriba, with manual review of supporting reads and gene coverage for clinical interpretation. The Targeted RNA Sequencing Panel detected HMGA2::GRIP1 fusion within the tumor and was annotated using transcripts ENST00000403681.2 (*HMGA2*) and ENST00000543172.1 (*GRIP1*) (Figure 3).

## Discussion

Microscopically, lipoblastomas harbor a varying proportion of bland-appearing primitive mesenchymal spindle cells, mature adipocytes, and lipoblasts arranged in a well-circumscribed lobular pattern and set in a variably myxoid stroma with increased thin-walled vessels [7]. A zonation pattern with the mature fat situated in the center and the spindle mesenchymal cells present at the periphery is a commonly described feature in lipoblastoma. The presence of such a zonation pattern in our case was a significant clue that raised suspicion of our lipoblastoma diagnosis.

While *PLAG1* immunohistochemistry has been regarded as cost-effective in diagnosing lipoblastomas [8], there is very little literature on its performance compared to molecular and cytogenetic studies. However, other immunostains that have been expressed to varying degrees in lipoblastoma were also helpful in our case, including variable expression of *CD34* and desmin.

With *PLAG1* rearrangement being the most common genetic alteration in lipoblastoma, multiple studies have identified the different fusion partners involved in its pathogenesis, including *COL1A2, COL3A1, HAS2, RAD51L1, RAB2A, BOC, CHCHD7, SRSF3, HNRNPC, PCMTD1, EEF1A1, YWHAZ, CTDSP2, PP2R2A, DDX6, KLF10, KANSL1L*, and the recently reported *PI15* and *ZEB2* genes [2,9]. The N-terminal of the *PLAG1* gene fusion partner in these cases binds to exon 2 or exon 3 of the *PLAG1* gene, causing increased transcription of the fusion gene. *PLAG1* copy number gain is also a common feature in lipoblastoma, whereby the tumor harbors one or more extra copies of chromosome 8, with or without concurrent rearrangement of 8q11-q13 [10].

On the other hand, *HMGA2*-rearranged lipoblastomas are very rare, with the three gene fusion partners reported thus far being *EP400*, *FGD6*, and *GSN* genes [5,6]. *GRIP1* as a gene fusion partner has not been previously reported in lipoblastoma. Our case represents a novel gene fusion partner to be reported in *HMGA2*-rearranged lipoblastomas.

*HMGA2* and *PLAG1* are both transcription factors involved in cell growth, proliferation, differentiation, and death. *PLAG1*, triggered by *IGF2* and its cognate receptor IGF1R, results in the activation of the MAPK signaling pathway, with *IGF2* stimulating the proliferation of adipocyte progenitor cells, thus leading to transcriptional up-regulation and neoplastic proliferation in lipoblastomas [11].

The function of *HMGA2* in lipogenic tumors has been well described in the literature [12]. Studies have shown that the loss of the last two exons of *HMGA2* grants the gene its transformative capability [4,13]. Sun et al. [14] showed that the level of *HMGA2* is highly regulated during adipogenesis by let-7, and that knockdown of *HMGA2* by miRNA inhibits both clonal expansion and the transition to terminal differentiation. Henriksen et al. [15] concluded that *HMGA2* overexpression keeps the mesenchymal stem-like cells in a proliferative state, preventing differentiation into adipocytes, and noted that the presence of *HMGA2* gene rearrangement is not inconsistent with the diagnosis of lipoblastoma, which is a lipogenic tumor comprised of adipocytes in different differentiation stages.

Glutamate receptor interacting protein 1 (*GRIP1*), an α-amino-3-hydroxy-5-methyl-4-isoxazolepropionic acid receptor (AMPAR)-binding protein, is involved in long-term potentiation (LTP) and learning and memory by regulating the trafficking and synaptic targeting of AMPARs, whereby the PDZ domain-containing protein of *GRIP1* specifically binds to the C termini of AMPA receptor subunits, resulting in the synaptic targeting of these receptors [16]. *GRIP1* is expressed early in embryonic development before the expression of AMPA receptors, with its highest expression levels present in the cerebral cortex, hippocampus, and olfactory bulb. Deletion of *GRIP1* in neurons blocks synaptic AMPAR accumulation induced by glycine-mediated depolarization [17]. HMGA2::GRIP1 fusion has been reported, to our knowledge, in one conventional lipoma with classic features [18].

The differential diagnosis of lipoblastoma includes myxoid liposarcoma, as both entities harbor myxoid, vessel-rich stroma; however, *DDIT3* gene rearrangement, which is a characteristic feature of myxoid liposarcoma, was not identified in our case, thus essentially excluding such a diagnosis. Lipoma with myxoid change also enters the differential diagnosis, especially since *HMGA2* fusions are commonly described in lipomas, and a similar HMGA2::GRIP1 fusion has been identified in one conventional lipoma thus far [18]. However, myxoid change was not seen in this aforesaid lipoma, which in fact harbored classic features and was comprised entirely of mature white fat, unlike our case, which was myxoid and vessel-rich in nature. Additionally, the zonation pattern in our case and the dual variable positivity for CD34 and desmin would strongly argue against the diagnosis of lipoma with myxoid change. Lipoblastoma-like tumor, although shares morphologic resemblance to lipoblastoma, is excluded based on the presence of *HMGA2* fusion, the variable desmin staining and the tumor location being in the chin; all of which are strong evidence against such an entity, as all reported cases of lipoblastoma-like tumor lack the above findings [19,20]. The increased stromal vascularity, the morphologic zonation pattern, and the presence of *HMGA2* fusion essentially excluded a spindle cell lipoma, an entity that is commonly characterized by monoallelic 13q14 deletion.

Our case highlights a novel gene fusion partner in lipoblastoma, thus broadening the molecular spectrum of such an entity. This finding increases awareness and prevents misclassification with other mimicking lipogenic tumors. However, given the rare nature of this gene fusion, the authors note that more future studies and additional research are needed to better characterize such cases.

## Conclusions

To conclude, we present a rare case of classic lipoblastoma arising in the chin of a 51-year-old female with an associated *HMGA2::GRIP1 *fusion, thus expanding the spectrum of gene fusion partners in* HMGA2*-rearranged lipoblastomas. This gene fusion is a novel finding in lipoblastoma and presents new evidence of the genetic heterogeneity of lipogenic tumors. Overall, our case expands the molecular spectrum of lipoblastomas beyond *PLAG1* gene rearrangements, thereby suggesting that lipoblastomas may be more molecularly heterogeneous than previously appreciated.
